# Expanding the diversity of DNA base modifications with *N*^6^-methyldeoxyadenosine

**DOI:** 10.1186/s13059-016-0874-7

**Published:** 2016-01-13

**Authors:** Kate D. Meyer, Samie R. Jaffrey

**Affiliations:** Department of Pharmacology, Weill Cornell Medical College, Cornell University, New York, NY 10065 USA

## Abstract

Vertebrate DNA is subjected to epigenetic base modifications that have been thought to be limited to methylated and other modified forms of cytidine. A recent study shows that methylation of adenine to form *N*^6^-methyladenine is a rare but readily detectable modification that can be mapped to distinct genomic sites in vertebrates.

## Background

Epigenetic modifications expand the information content of DNA and have long been known to exist in the genomes of diverse organisms. The most well-studied base modification in vertebrates and higher eukaryotes is methylation of the C-5 position of cytosine residues, which forms the 5-methylcytidine (m5C) nucleotide. m^5^C is often detected at CpG dinucleotides where it influences transcription by recruiting repressive m^5^C-binding proteins or preventing the binding of transcription factors [1]. Importantly, cytidine methylation is a reversible modification, and both methylation and demethylation pathways contribute to dynamic regulation of m^5^C signatures which control both developmentally regulated and activity-dependent gene expression programs [1]. Demethylation of m^5^C residues has been shown to involve the formation of oxidized intermediates, such as 5-formylcytidine and 5-hydroxymethylcytidine, which also contribute to gene expression control. Thus, the cytidine nucleotide has long been considered to be the major site of DNA base modifications contributing to transcriptional regulation in vertebrates and other eukaryotes.

Unlike higher eukaryotes, many bacteria are known to contain additional DNA base modifications. These include C-4-methylated cytosine (4mC), as well as methylated adenine (*N*^6^-methyladenine, or 6 mA). The methylated base, 6 mA, or the corresponding nucleotide *N*^6^-methyldeoxyadenosine (m^6^dA), is an important component of the restriction/modification system used to defend against bacteriophage invasion. This system uses the methylation of host cell DNA to protect it against cleavage by restriction endonucleases, while enabling invading, unmethylated genomic material to be cleaved. In addition to its role in host defense pathways, m^6^dA is also an important regulator of DNA replication, repair, and transcriptional control in prokaryotes [2].

## A new addition to the vertebrate epigenome

Although m^6^dA is a readily detectable feature of bacterial genomes, it has been more difficult to definitively establish its presence in the genomes of higher organisms. This contrasts with mRNA, where mapping of the ribonucleotide equivalent of m6dA, *N*^6^-methyladenosine (m^6^A), has identified m6A sites in thousands of mammalian mRNAs [3]. Studies aimed at detecting m^6^dA in the DNA of higher organisms [4] have failed to uncover evidence for the existence of this modification. Although such studies formed the basis for the belief that mammalian DNA is devoid of m^6^dA, they were done over 40 years ago and were hampered by low sensitivity (limit of detection approximately 0.01 %) In contrast, more recent studies have challenged the notion that eukaryotic DNA does not contain m^6^dA. In 2006, Wion and colleagues used liquid chromatography coupled with mass spectrometry to interrogate the mouse genome for the presence of m^6^dA and detected very low levels of m^6^dA (fewer than 1 m^6^dA per 10^6^ nucleotides) [5]. Although highly sensitive, such approaches can also lead to artifacts since trace amounts of bacterial contamination in genomic preparations can result in the detection of m^6^dA due to the high levels of m^6^dA in their genome. Indeed, bacterial contamination is commonplace in mammalian culture systems, and bacteria are either commensal organisms, or part of the diet of lower organisms such as *Caenorhabditis elegans* and *Drosophila.*

A major advance came with the development of m^6^dA mapping techniques in invertebrate organisms. Three recent studies used global m^6^dA profiling methods to identify m^6^dA in the genomes of *Chlamydomonas reinhardtii*, *Drosophila melanogaster*, and *C. elegans*, with levels of m^6^dA ranging from 0.4 % to 0.001 % of total adenine residues within these genomes [6–8]. Since these methods identified m^6^dA within a genomic sequence, the m^6^dA could be definitively assigned to the invertebrate genome rather than caused by bacterial contamination. These studies have been an important advance in our understanding of the repertoire of epigenetic modifications in higher organisms.

The outstanding question was whether m^6^dA is found in higher organisms, including humans. A recent study from Gurdon and colleagues [9] provides the first analysis of vertebrate m^6^dA residues genome-wide. The study, published in *Nature Structural & Molecular Biology*, examined the genomes of frogs, mice, and human cells using ultra-high-performance liquid chromatography with tandem mass spectrometry and revealed low levels of m^6^dA in all three genomes (approximately 1 m^6^dA for every 1.2 × 10^6^ deoxyadenosine residues, or 0.00009 % of deoxyadenosine residues).

The authors then went on to globally profile m^6^dA distribution within *Xenopus* and mouse genomes using an m^6^dA antibody-based DNA immunoprecipitation (DIP) method. In brief, this method involves immunoprecipitating DNA fragments that contain m^6^dA using a m^6^dA-specific antibody. Their analysis revealed a large number of reads that cluster to form m^6^dA peaks in these genomes (approximately 20,000–50,000 depending on the tissue). Notably, only a small number of m^6^dA peaks were located within genes (approximately 7–21 %). Peaks within genes were largely excluded from exons, but a higher number were located within intronic regions. In addition, the authors observed a relative paucity of m^6^dA sites immediately after transcription start sites (TSSs), which contrasts with the marked enrichment of m^6^dA within this region in *C. elegans* [8].

The authors validated their results by repeating the global mapping studies in *Xenopus* using two additional m^6^dA antibodies. There was a high degree of overlap of individual m^6^dA peaks as well as overall m^6^dA distribution among all three antibodies tested, indicating that the DIP-seq mapping technique is likely detecting valid m^6^dA sites. When comparing m^6^dA sites across different *Xenopus* tissues, there were subsets of m^6^dA sites that were common to two or more tissues, but others that were unique. This may indicate tissue-specific m^6^dA patterns, but warrants further investigation to rule out sample-to-sample variability. Similarly, comparison of m^6^dA peaks in frog and mouse genomes revealed some peaks that overlapped and others that were distinct, which may be due to different tissues examined in each species (testes, fat, and oviduct in frog and kidney in mouse) or to species-specific methylation patterns. Additional detailed analyses of m^6^dA distribution across various tissues and in diverse species will likely lend further insights into the tissue-/cell type-specific distribution of m^6^dA as well as the consistency of m^6^dA sites across species.

The overall pattern of m^6^dA localization observed in the Gurdon study, as well as in the other recent m^6^dA profiling studies, provides the first insights into the potential function of m^6^dA. Although Gurdon and colleagues observe a slight enrichment of m^6^dA peaks just upstream of TSSs, the most defining feature of m^6^dA observed in this region is an absence of m^6^dA just after the TSS. In contrast, Greer et al. report no clear distribution of m^6^dA near genes in *C. elegans* [8], whereas m^6^dA is enriched at and just after TSSs in *Ch. reinhardtii* [6]. These distinct distribution patterns might indicate different roles for m^6^dA in vertebrates than in lower organisms. The fact that m^6^dA exhibits variable localization around TSSs further suggests potential functions for this modification in transcriptional regulation. Indeed, m^6^dA in *Ch. reinhardtii* is associated with actively transcribed genes [6]. However, further studies will be necessary to determine whether m^6^dA is repressive or permissive for transcription in vertebrates. Such analyses will also be useful for understanding how relevant functional studies in lower organisms are likely to be for understanding m^6^dA in vertebrates.

## Moving forward: m^6^dA regulatory pathways and functional insights

A major priority right now for m^6^dA research is to uncover the function of m^6^dA, a task which will be facilitated by a more detailed understanding of the readers and writers of this mark. A putative m^6^dA methyltransferase, DAMT-1, has been identified in *C. elegans* [8], although the closest vertebrate homolog of this protein (METTL4) has not yet been explored for m^6^dA-forming potential. Notably, Gurdon and colleagues identify an AG-rich motif which was enriched in m^6^dA peaks in *Xenopus*. Two AG-containing motifs were also detected at m^6^dA sites in *C. elegans* [8], suggesting that the m^6^dA methyltransferase machinery in these organisms might share a similar recognition sequence. However, efforts to identify m^6^dA motifs in other higher eukaryotes, such as mice and flies, have failed to identify consistent consensus sequences [7, 9], suggesting the existence of other mechanisms for m^6^dA formation in addition to simple sequence recognition elements.

**Fig. 1:**
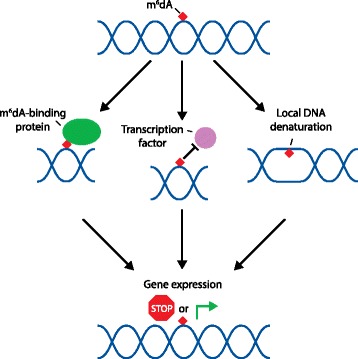
Methylated deoxyadenosine (*m* ^*6*^ *dA*) residues in DNA have the potential for multiple functions within the eukaryotic genome. m^6^dA residues might recruit m^6^dA-binding proteins (*left*), or m^6^dA might prevent the binding of transcription factors (*middle*). In addition, because *N* ^6^ methylation of adenosines can influence Watson:Crick base pairing, m^6^dA might act by inducing local DNA denaturation (*right*). Any or all of these possible functional roles for m^6^dA have the potential to influence transcription or other modes of gene expression. Whether m^6^dA acts primarily by repressing or promoting gene expression remains to be determined

How does m^6^dA regulate DNA function? One possibility is that m^6^dA mediates the recruitment of transcription factors and/or m^6^dA binding proteins (Fig. 1). Although readers of m^6^A in RNA have been identified, proteins that specifically recognize methylated adenosine residues in DNA remain to be uncovered. The major epigenetic mark in vertebrates, m^5^C, is known to act in part by recruiting m^5^C-binding proteins such as MECP2 [10], and it is possible that m^6^dA functions in an analogous manner. Thus, a better understanding of the proteins that read and write the m^6^dA mark will be necessary to perform knockout studies that are likely to reveal m^6^dA function.

Another possibility is that m^6^dA functions to destabilize DNA duplexes. Although m^6^dA forms standard Watson:Crick base pairs with thymidine, the base pairs between m^6^dA and thymidine are less stable than canonical adenosine:thymidine base pairs. Thus, m^6^dA may facilitate DNA unwinding or the open state of DNA needed for transcription initiation and other processes (Fig. 1).

An intriguing feature of m^6^dA across diverse organisms is that its abundance is markedly decreased in more evolved organisms. Could this be due to the evolution of active m^6^dA demethylation pathways? In other words, m^6^dA could occur frequently, but its rapid removal may account for its low overall abundance in the genome. NMAD-1, a homolog of the m^6^A RNA demethylase ALKBH5, has shown evidence of m^6^dA demethylation in *C. elegans* [8]. Interestingly, the *Drosophila* homolog of Tet (called Dmad), which normally functions as a m^5^C demethylase in vertebrates [11], was identified as a m^6^dA demethylase in flies [7]. Its potential as a m^6^dA demethylase in vertebrates, however, remains to be tested.

In addition to uncovering m^6^dA demethylases in vertebrates, it will be important to determine whether these enzymes produce demethylated intermediates, as is seen with the Tet-catalyzed production of 5-hydroxymethylcytidine (hm^5^C) from m^5^C residues in eukaryotic DNA [11]. Thus, there remains the possibility that the repertoire of modified nucleotides expands beyond m^6^dA. Such intermediate forms of modified adenine, if present, might also perform unique functions in regulating gene expression and thus represent important areas of future exploration.

## Abbreviations

6 mA: Methylated adenine
DIP: DNA immunoprecipitation
m^5^C: 5-Methylcytidine
m^6^A: *N*^6^-methyladenosine
m^6^dA: *N*^6^-methyldeoxyadenosine
TSS: Transcription start site

## References

[1] Jones PA (2012). Functions of DNA methylation: islands, start sites, gene bodies and beyond. Nat Rev Genet.

[2] Løbner-Olesen A, Skovgaard O, Marinus MG (2005). Dam methylation: coordinating cellular processes. Curr Opin Microbiol.

[3] Meyer KD, Saletore Y, Zumbo P, Elemento O, Mason CE, Jaffrey SR (2012). Comprehensive analysis of mRNA methylation reveals enrichment in 3′ UTRs and near stop codons. Cell.

[4] Gunthert U, Schweiger M, Stupp M, Doerfler W (1976). DNA methylation in adenovirus, adenovirus-transformed cells, and host cells. Proc Natl Acad Sci U S A.

[5] Ratel D, Ravanat JL, Charles MP, Platet N, Breuillaud L, Lunardi J (2006). Undetectable levels of N6-methyl adenine in mouse DNA: cloning and analysis of PRED28, a gene coding for a putative mammalian DNA adenine methyltransferase. FEBS Lett.

[6] Fu Y, Luo GZ, Chen K, Deng X, Yu M, Han D (2015). N6-methyldeoxyadenosine marks active transcription start sites in chlamydomonas. Cell.

[7] Zhang G, Huang H, Liu D, Cheng Y, Liu X, Zhang W (2015). N6-methyladenine DNA modification in Drosophila. Cell.

[8] Greer EL, Blanco MA, Gu L, Sendinc E, Liu J, Aristizábal-Corrales D (2015). DNA methylation on N6-adenine in C. elegans. Cell.

[9] Koziol MJ, Bradshaw CR, Allen GE, Costa AS, Frezza C, Gurdon JB (2015). Identification of methylated deoxyadenosines in vertebrates reveals diversity in DNA modifications. Nat Struct Mol Biol.

[10] Lewis JD, Meehan RR, Henzel WJ, Maurer-Fogy I, Jeppesen P, Klein F (1992). Purification, sequence, and cellular localization of a novel chromosomal protein that binds to methylated DNA. Cell.

[11] Tahiliani M, Koh KP, Shen Y, Pastor WA, Bandukwala H, Brudno Y (2009). Conversion of 5-methylcytosine to 5-hydroxymethylcytosine in mammalian DNA by MLL partner TET1. Science.

